# Using Deep Dive Methodology to Investigate an Increased Incidence of Hospital-Acquired Avoidable Category 2 and 3 Pressure Ulcers

**DOI:** 10.3390/healthcare7020059

**Published:** 2019-04-08

**Authors:** Martyne Horton-Jones, Emma Marsh, Sian Fumarola, Helen Wright-White, Wilfred McSherry, Trish Rowson

**Affiliations:** 1 The University Hospitals of North Midlands NHS Trust, Newcastle Rd, Stoke-on-Trent ST4 6QG, UK; Emma.Marsh@uhnm.nhs.uk (E.M.); sian.fumarola@uhnm.nhs.uk (S.F.); h.wright-white@keele.ac.uk (H.W.-W.); W.McSherry@staffs.ac.uk (W.M.); Trish.Rowson@uhnm.nhs.uk (T.R.); 2 Department of Nursing, School of Health and Social Care, Staffordshire University, Blackheath Lane, Stafford ST18 0YB, UK; 3 VID vitenskapelige høgskole, Haraldsplass Bergen, Ulriksdal 10, 5009 Bergen, Norway

**Keywords:** pressure ulcers, deep dive, frailty, quality, safety, patient experience, older people

## Abstract

**Background**: Between October 2017 and March 2018, the Trust experienced significant winter pressures and an increase in category 2 and 3 hospital-acquired avoidable pressure ulcers. This review aimed to investigate the causal factors of this increase. **Methods**: A ‘Deep Dive’ review of 37 cases was undertaken in three stages: (i) assurance; ensure the increase was not due to insufficient equipment; (ii) collation of relevant data, including age, length of time in A&E, bed surface, number of internal moves; (iii) analysis identifying factors that might account for the observed increase. **Findings**: Age combined with prolonged length of time in A&E, being nursed on a trolley followed by three or four internal moves were observed in patients who developed pressure ulcers. Patient age was observed as a key factor, with those over 80 years experiencing pressure ulcers more frequently. **Conclusion**: The small size of this data suggests a need for the greater awareness of frailty issues in older people, timely assessment and intervention to prevent a chain of detrimental factors might be key to reduce and prevent hospital-acquired avoidable pressure ulcers. Recommendations for immediate action, education and future research have been made to the Trust Quality and Safety Committee.

## 1. Introduction

Ensuring the safety of all patients is a major priority for all healthcare providers. The provision of healthcare is complex, involving many systems, processes, interactions and personnel, meaning that there are many stages along the patient’s pathway where situations can occur that could compromise their safety and well-being. This is especially the case for those admitted into hospital who may be considered frail and vulnerable, living with multiple co-morbidities. One area where there has been a tremendous amount of investment is in preventing patients from developing hospital-acquired avoidable pressure ulcers [1]. Reporting on data collected between April 2013–March 2014) found the percentage of avoidable PUs was less than a third of the 95% figure that has been previously quoted [2]. Despite questions surrounding the accuracy of reporting hospital-acquired avoidable pressure ulcers, the impact of acquiring such tissue damage (whether avoidable/unavoidable) can have serious consequences on the patient’s recovery and mortality. As has been debated, the importance of timely assessment, screening and ultimately intervention is of paramount concern in the prevention of pressure ulcer formation [3].

### 1.1. Local Context

The University Hospitals of North Midlands NHS Trust (UHNM) is committed to the reduction of hospital-acquired avoidable pressure ulcers. In order to meet this commitment, the Trust is engaged with the international “Stop the Pressure” initiative (The European Pressure Ulcer Advisory Panel (EPUAP), 2018) [4] with the Tissue Viability Team providing on-going staff education and updates on the latest guidance and research. UHNM has also implemented several Trust-wide campaigns. These include for example, ‘Happy Heels’, ‘Five Moments of Pressure Ulcer Prevention’ and ‘Catch the Blanch’. The Trust has also held a ‘Stop the Pressure’ week-long mobile staff event across both hospital sites and hosts extremely well-attended annual Tissue Viability Conferences. Locally, Link Nurses and Quality Nurses focus education, updates and quality improvement projects on the specific needs of their clinical areas. Some of the many examples of these would include, Trauma Orthopedics who ran a year-long focus on skin bundle completion, the Elderly Care Wards who developed a daily Body Mapping Tool for accurate documentation of frail skin and the operating departments who designed documentation to monitor patient’s pressure areas during their time in surgery. All these interventions have focused on specific aspects of pressure ulcer prevention as identified through root cause analysis. 

Alongside this, the UNHM Tissue Viability Team have been continuously engaged in the review, evaluation and procurement of an extensive variety of pressure-relieving devices. This has resulted in high-specification dynamic foam mattresses, silicone-pressure redistribution pads and a comprehensive range of heel offloading devices being made available and utilized by the wards and clinical areas across the Trust. These approaches have enabled the UHNM to meet the requirements of the Clinical Commission Group (CCG) Commissioning for Quality and Innovation (CQUIN) measures. This resulted in a reduction of 46% in reported pressure ulcers during the period 2013–2014; however, pressure ulcers are not included in the current CQUINS.

Over the following three financial years, UHNM has not been able to maintain this downward trajectory. The last financial year, 2017–2018, saw a downward trend in the year on year growth. However, during the winter months, between October 2017 and March 2018, where organization pressures were at their highest, reporting rose dramatically. As a result, the year ended with an overall increase of 18%.

### 1.2. External Factors

During the winter months, more people with complex needs require admission to hospital and demographic changes related to an ageing population and increases in obesity and diabetes will affect vulnerability to pressure ulceration. A report from the Health Foundation (2018) [5] stated that in 2015/6 one in three emergency patients admitted for overnight stay had five or more health conditions compared to 1:10 in 2005/06. Other factors include the integration of County Hospital in November 2014, an increase in bed numbers at the Royal Stoke site with additional beds at the Haywood Hospital to accommodate them.

### 1.3. Aims

In response to an upward trend in the incidence of hospital-acquired avoidable pressure ulcers (see Table 1) (especially category 2 and 3) across the organization, the UHNM Quality and Safety team elected to review this period of increased organizational pressure with the aim of identifying whether there were any factors that may have precipitated or contributed to this upward trend.

By focusing on the cases where Root Cause Analysis (RCA) determined these incidents/pressure ulcers as avoidable, the team were keen to maximize the opportunities for learning and to inform future practice and prevent recurrence. Root Cause Analysis (RCA) is a structured method used to analyse serious adverse events. Initially developed to analyse industrial accidents, RCA is now widely deployed as an error analysis tool in health care. Sherwin (2011, p.28) [6] provides a useful explanation of Root Cause Analysis highlighting some of the key issues in using this as a management tool. She states that “Using root cause analysis, clinical teams can examine all the steps related to a harmful incident, or a near miss, identify the root (the original cause or causes), and create solutions to prevent repetition”. Therefore, RCA was deemed to be appropriate in the context of conducting this service evaluation.

It was noted that, during this specific timeframe, UHNM experienced an increase of 5.97% in emergency admissions and proportionally, an increase of 5.92% in admissions of frail elderly patients over the age of 70 years. However, though notable, the increase in admission numbers alone does not fully explain the increase in hospital-acquired avoidable pressure ulcers during this period and fails to assist the Trust to prepare for future periods of increased organisational pressure.

It was observed that the increase in activity also saw a corresponding rise in reported incidents in this period, which was 82%. Whilst these statistics illustrate an increase in both incidents and admissions, they do not explain whether there is a causal relationship between the two, or the increase in hospital-acquired avoidable pressure ulcers at 29% during this period and, thus, fail to assist the Trust to prepare for future organisational pressures.

Therefore, the aims of this evaluation were to:

- Undertake a ‘Deep Dive’ into the case notes of patients who developed a hospital-acquired avoidable pressure ulcer (category 2 and 3) during the period, October 2017 to March 2018.
- Explore whether there were any specific factors or variables that may account for or explain the increase in patients developing grade 2 and 3 pressure ulcers during this period.
- Make recommendations both in the short and long term to prevent this situation from occurring in the future.

## 2. Materials and Methods

### 2.1. Participants

Between October 2017 and March 2018, 37 patients were determined to have developed hospital-acquired avoidable pressure ulcers at UHNM. Table 2 provides an overview of the demographics and some of the admission variables included in the evaluation. 

**Figure 1:**
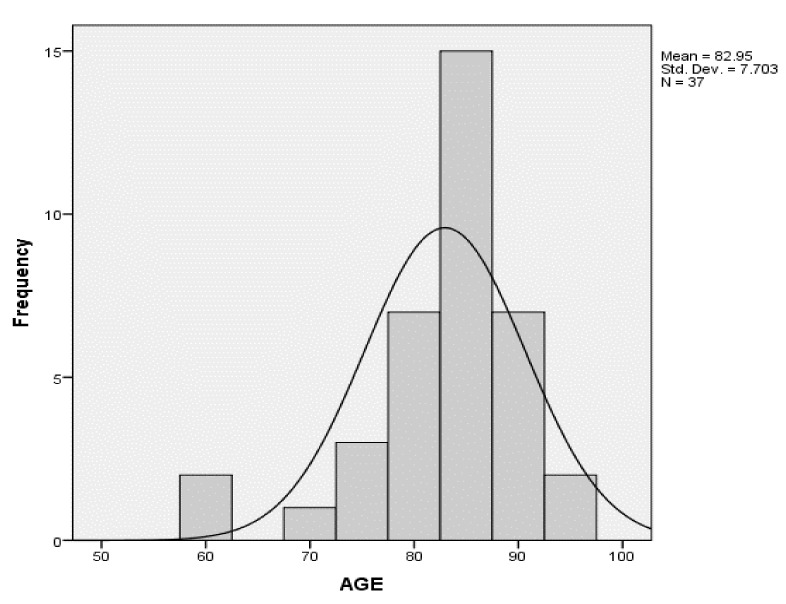
Age distribution.

The sample population consisted of 18 males and 19 females, for which the mean age of males was 83.25 years (SD = 5.64 years) and the mean age of females was 81.7 years (SD = 9.85 years). Figure 1 shows the distribution of age was approximately equal across males and females and the age distribution was approximately normal, showing a spike in the 81–90-year-old category.

### 2.2. Procedure

The evaluation was exploratory in design employing a ‘Deep Dive’ methodology which is a technique used in service improvement, enabling a team to rapidly immerse themselves into a specific situation to gain a ‘deeper’ understanding of a problem and gain insights with the aim of generating a solution. The Good Governance Institute (2016, p.5) [9] suggests a Deep Dive may be appropriate when the following factors exist:

- an investigation of something gone awry, not understood or where independent assurance is lacking;
- something more than usual performance management, audit, assurance;
- a limited exercise producing understanding, conclusions and actions.

From the above, this methodology suited this specific evaluation, since it allowed the team to focus upon the increasing prevalence of pressure ulcers during a specified period. The Deep Dive evaluation comprised three interrelated stages:

#### 2.2.1. Stage 1: Assurances

Prior to undertaking the case reviews, the team (MH-J, EM) reviewed the Root Cause Analysis findings of the hospital-acquired avoidable pressure ulcers. This provided confirmation and assurance that there had been no changes of policy, procedure or clinical practice that could account for the increased incidents. They then confirmed that the increase in hospital-acquired avoidable pressure ulcers during the period was not simply the consequence of not having enough resources, such as the dynamic mattresses and heel offloading devices to accommodate the needs of the increased number of patients. This was achieved through liaison with our pressure mattress store and supplies department who confirmed that despite a swell in admissions, UHNM maintained, and staff could access, sufficient stock without significant delay at all times to meet increased patient numbers. Similarly, the RCA review also confirmed that a lack of appropriate preventative equipment did not feature as a factor in the development of pressure ulcers during the period under review. 

#### 2.2.2. Stage 2: Identification and Selection of Cases

The review began with the selection of cases to be included and initially all the category 3 hospital-acquired avoidable pressure ulcers, identified via Root Cause Analysis, in the period October 2017 to March 2018, were selected for inclusion. 

The case note review was then extended to include category 2 hospital-acquired avoidable pressure ulcers because, although not classified as a Serious Incident (SI) (multiple category 2 ulcers may be reportable as SI), the purposes of this report is to examine the underlying factors behind the increase in the development of pressure damage, which necessitated the inclusion of pressure ulcers regardless of category status. In addition, the ‘category’ of damage is greatly influenced by both the patient’s general condition and their clinical management after development.

Therefore, since the aim was to focus on understanding the increase in incidents, rather than severity, all avoidable category 2 and 3 pressure ulcers were included. This is consistent with the approach adopted by UHNM who have elected to investigate all category 2 and above pressure ulcers even though only pressure ulcers that meet the criteria for a SI should be reported to the clinical commissioning group and are externally reportable (NHS England,2015) [10].

#### 2.2.3. Stage 3: Individual Case Review 

This review involved in-depth scrutiny and analysis of individual cases during the specified period focusing on several criteria. A comprehensive list of the factors/variables included in the evaluation are provided in Box 1. The aim of using these broad criteria was to avoid the already well-researched patient risk factors (Coleman et al., 2013) [11] and instead, alongside the location and size of the pressure ulcer, focus on the patients’ reason for admission, their organizational journey and the initial form of presentation. Adopting this wider approach may determine if there were any patterns or specific factors surrounding the development of pressure ulcers and/or which would explain why these avoidable pressure ulcers were not prevented during the 6 months of increased incidence. 

The case note review initially focused on 50 cases reported and determined via root cause analysis presented at the RCA Panel to have been deemed hospital-acquired avoidable pressure ulcers. Due to challenges in obtaining the case notes for all 50 cases, some notes being in the process of scanning and, therefore, not available for review, this number was reduced to 37 cases.

Box 1 **Factors/variables included in the evaluation.**

Age

DoB

Gender

Timespan to PU development from admission (days)

Sequential clinical area (moves)

Cognitive impairment

Compliance

Primary diagnosis

Secondary diagnosis

A&E attendance

A&E in hours—time in A&E department or time of arrival

Secondary admission portal

A&E surface, i.e., A&E trolley/stryker trolley/bed with static foam mattress/bed with dynamic mattress

Size of pressure ulcer

Admission body map completion (time + skin condition)

Waterlow risk assessment

Deep tissue injury (DTI)

End of life care (EOL)

Blister

Moisture present to skin

#### 2.2.4. Ethical Considerations

The project was reviewed internally, utilizing the on-line Health Research Authority (HRA) tool and since it was anonymous, retrospective data that could not be generalized was not deemed to be research and, therefore, did not require ethical approval. Consequently, there were no ethical issues associated with this ‘Deep Dive’ as it was a service evaluation and part of ensuring the quality and safety of patients in receipt of care at UHNM.

#### 2.2.5. Data Analysis

After individual case reviews, all the data pertaining to each of the individual factors were collated and analysed to establish whether there were any indications of potential underlying connections. The data set was small and contained only patients who had developed pressure ulcers, which limited the type of analysis that can be robustly undertaken. It was not possible to develop a predicative model for pressure ulcer development, because, in order to do this, one must have both patients who developed and did not develop pressure ulcers. In addition, one must have a minimum of 10 cases per predictive variable. Consequently, due to only having 38 cases of patients with pressure ulcers, the development of a predictive model was not feasible. Similarly, the data contained only one group of patients, and so the difference between groups, i.e., between those who developed and did not develop a pressure ulcer, could not be tested for significance. Any testing for differences between males and females would run the risk of a Type I error, since the group sizes would be very small. Therefore, only descriptive statistics were used to summarise and report the data, which will inform more robust future research and provide sufficient information to generate a formal sample size calculation.

## 3. Results

This section provides the findings of the initial investigation of the data. The report does not present inferential interpretations due to the limitation of the data discussed above. The focus is on the identification of any potential association between variables that may contribute to the explanation of the increase in category 2 and 3 pressure ulcers during this period. Further robust research needs to be conducted in order to establish how accurately these potential factors predict the occurrence of a pressure ulcer but, at this time, limited data meant such statistical modelling was not possible.

### 3.1. Admission and Patient Journey

Of the 37 cases, 21.6% were admitted through County Hospital, whilst 78.3% of cases were admitted through Royal Stoke University Hospital. Of these, 86.4% of patients were admitted to A&E, whilst only 13.5% were not admitted through A&E. Of those patients who were admitted via A&E, 75% went through a secondary admission portal such as the Acute Medical Unit (AMU) or the Frail Elderly Admission Unit (FEAU).

### 3.2. Time in A&E

**Figure 2:**
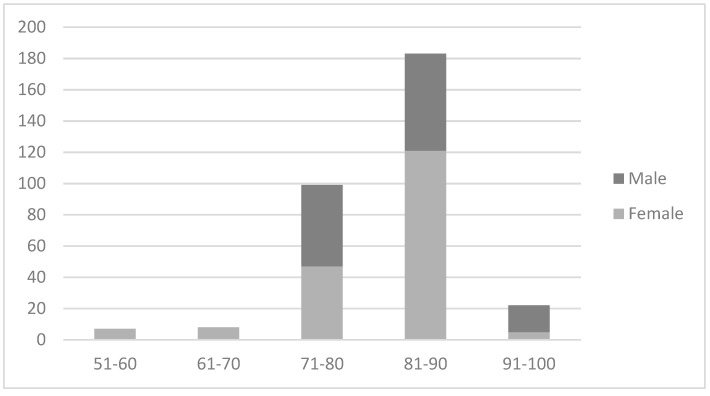
Hours in A&E by age and gender.

An analysis of the time spent in A&E illustrated that whilst, on average, females spent longer in A&E than males, this was not statistically significant. The exact reasons for this cannot be ascertained from this preliminary evaluation. In addition, Figure 2 (below) shows that patients aged 81–90 years spent the longest time in A&E—the majority were female, spending a total of 183 hours in total compared to the age group 71–80 years, who spent a total of 99 hours in A&E. 

There was, however, no significant correlation found between age and number of hours in A&E (*r* = 0.09, *p* = 0.608). However, this is a very small number of purposefully selected cases and does not exclude the possibility that an association might exist between age and time in A&E in a more representative sample. Further analysis revealed that there was wide variation in the number of hours this group spent in A&E and the precise reasons for the extended wait requires further exploration.

The finding that, on average, females spent longer in A&E than males was not found to be significant. An independent samples t-test was conducted to compare time in A&E between males and females. There was no significant difference in the time spent in A&E between males (m = 32.33, SD = 63.94) and females (m = 43.16, SD = 56.65) (t(35) = −0.546, *p* = 0.589. 95%CI = −51.1, 29.4). However, one reason for this result is the very small sample size.

The majority of these patients were also found to be aged between 71 and 90 years. As the advanced years of these patients increased, their risk of pressure damage did so too, according to Waterlow (2005) [12]—a link with gender was also suggested. These patients are also likely to have been frail. These results might suggest that those who spent longer in A&E are more at risk of developing a pressure ulcer. However, this evaluation did not have available data relating to patients who (i) spent less time in A&E and (ii) did not develop a pressure ulcer; therefore, this conclusion cannot be made without further research.

The Royal College of Nursing (2018) [13], in discussing frailty, states:

“Frailty occurs more often as people become older. Of people over 85 years of age about one in four is living with frailty and increasingly it is suggested that frailty needs to be thought of as a long-term condition.”

Therefore, based on the findings from this evaluation, many of these patients will fall into this classification given that, in addition to their advance years, by virtue of their need for admission, they also had an acute medical condition requiring intervention. 

Table 3, above, shows that during their spell in A&E, 35 patients progressed to develop a hospital-acquired avoidable pressure ulcer. Of these, eight (22.9%) did not have a body map, risk assessment and/or skin bundle completed appropriate to their predictable risk. The absence of a body map and/or skin bundle makes it difficult to confirm that these patients received appropriate preventative intervention. Where skin bundles were completed, they provide limited evidence of positional changes and only one patient had received heel offloading. 

Consequently, there appears to have been a failure of the normal pressure ulcer prevention process during this period of increased pressure. This may have been due to the number of patients requiring nursing care and/or the necessity to nurse patients in out or open-spaces (not in a cubicle or bay) which makes skin inspection challenging to achieve. However, it is not possible to confirm the location of these patients given the lack of documentation.

This apparent failure may be compounded by using trolleys in A&E. In the sample population, 74.3% had been cared for on a trolley in A&E and only 0.08% were in a bed, and 17% had no information recorded. Therefore, the majority of patients who developed a pressure ulcer were nursed on a trolley in A&E. 

**Figure 3:**
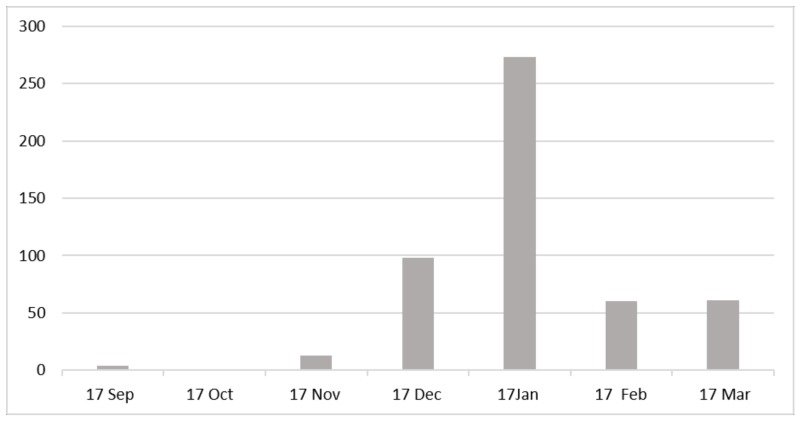
Number of A&E breaches by month.

Figure 3 illustrates the number of 12-h breaches over the period under investigation. In this initial investigation, the number of A&E breaches could not be matched to those patients identified with a hospital-acquired avoidable pressure ulcer. However, it should be noted that any patients who had a deep tissue injury (DTI) identified within 72 h of admission would not be captured in this review. This is due to NHS Midlands and East (2012) [14] guidelines that were in place during this period. These guidelines stated that any deep tissue injury identified within 72 h of admission was considered to have been present on admission and as such would not have been investigated as a hospital-acquired pressure ulcer. 

Although it is therefore not possible to match the number of breeches to the patients identified with a hospital-acquired pressure ulcer, the chart above does serve to highlight the additional pressure experienced by emergency portals that may have contributed to the lapse in the normal pressure ulcer prevention process. 

### 3.3. Development of a Pressure Ulcer

There appeared to be an approximately even split between the reporting of category 2 and category 3 pressure sores and amongst males and females (Table 4). 

#### Types of Injury

The analysis of the type of injury that occurred revealed that 62% of pressures ulcers occurred on heels, for both males and females, the next most common occurrence was the coccyx, in 21.6% of cases. 

A Chi-square test of independence was calculated comparing the location of pressure ulcers with the number of sequential clinical moves. No significant interaction was found (X^2^ (12) = 8.439, *p* = 0.750); however, 90% of cells in the analysis had less than the minimum expected count, suggesting that the results are not reliable.

**Figure 4:**
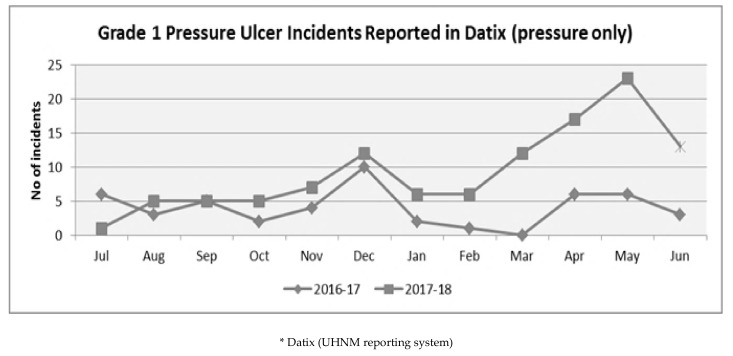
Grade 1 pressure ulcer incidents reported in Datix.

Of note is that 24.3% of non-heel pressure ulcers were incident reported as less than 1 cm × 1 cm. The reporting of small sized ulcers may be a reflection on the effectiveness of the ‘Catch the Blanch’ campaign, which centred around acting on small changes in colour and tissue profusion at the skin surface. Alongside this, the ‘Five Moments of Pressure Ulcer Prevention’ campaign focused staff on the importance of early identification, communication and the reporting of pressure ulcers. However, it is not possible to statistically confirm this, as such small sores tend to heal quickly once identified and clinically managed. Therefore, if not reported, they would not be likely to feature in the Root Cause Analysis conducted in previous periods. There is evidence, however, of an increase in reporting of Category 1 pressure ulcers since the education campaign (Figure 4). This would support the suggestion that staff are actively engaged in the reporting of pressure ulcers at a much earlier stage following the campaign and therefore would be equally likely to be identifying Category 2 + pressure ulcers at an earlier stage and smaller size. 

The average time from admission to the development of a pressure ulcer was found to be approximately 10 days for males and 9 days for females; this was not found to be a significant difference (t(35) = −0.394, *p* = 0.696, 95% CI = −15.8, 10.69). The variance in the time from admission to the development of pressure ulcers for both males and females suggests that there is no single common point when pressure damage has occurred in this small sample population. This is a limitation of the size of the data set, and a much larger sample would be needed to reduce this variance and give a more accurate indication of the average time from admission to the development of a pressure ulcer for most people. Further work and a bigger sample would be required to make any observations. 

As previously mentioned, due to NHS Midlands and East (2012) [14] guidance, any Deep Tissue Injuries (DTI) identified within 72 hours would not be captured in this table. Consequently, the average time to development may appear longer due to the exclusion. Therefore, a case review of all Deep Tissue Injuries coded as present on admission would be required to increase the accuracy of this information.

In addition, it was found that over 16 patients had three moves, and three patients had four moves, before a pressure ulcer developed or was detected and reported by clinical staff. Of the cases reviewed, 21.6% of pressure ulcers were first identified as suspected deep tissue injury (SDTI) “suspected deep tissue injury” is defined by the National Pressure Ulcer Advisory Panel, European Pressure Ulcer Advisory Panel and Pan Pacific Pressure Injury Alliance (2014, p.13) [15] as:

“Purple or maroon localized area of discolored intact skin or blood-filled blister due to damage of underlying soft tissue from pressure and/or shear. The area may be preceded by tissue that is painful, firm, mushy, boggy, warmer or cooler as compared to adjacent tissue. Deep tissue injury may be difficult to detect in individuals with dark skin tones. Evolution may include a thin blister over a dark wound bed. The wound may evolve and become covered by thin eschar. Evolution may be rapid exposing additional layers of tissue even with optimal treatment.”

Therefore, it cannot be assumed that the pressure damage necessarily occurred in the area in which it was identified. The damage may have begun in the emergency portals or even prior to admission. Without accurate documentation during this acute phase of the patient’s admission, it is not possible to confirm or refute this argument. However, it does reinforce the need for vigilance and timely assessment and pressure intervention in all our emergency portals. 

The argument that the pressure ulcer may have occurred at an earlier point during the patient’s hospital journey is supported by Farid et al. (2007) [16] who indicate that the time between the precipitating event (a period of prolonged pressure, for example the extended time in the A&E department lying on a trolley) and the occurrence of deep tissue damage can be anywhere between 24 hours and 7 days. Alternatively, given that as previously highlighted many did not have a body map, it may even have been possible that some of the damage to deep tissues may have begun before admission. 

In addition, in 24.3% of the cases reviewed, the pressure ulcers were first identified as blisters. This finding may also bring into question issues of manual handling between clinical areas/lateral transfer between surfaces because of the effects of friction and sheering on the skin. 

## 4. Discussion

The aim of this service evaluation was to explore possible factors and variables that may help explain the increase in observed hospital-acquired avoidable grade two and three pressure ulcers between October 2017 and March 2018. This evaluation comprised three stages each conducted in a robust and rigorous manner involving an in-depth case review and analysis. Due to the small sample size and limited inclusion, it is not possible to make inferences from these findings or generalise about the exact reasons or causal mechanism that have led to the increase in prevalence. However, the evaluation does shed some valuable light on identifying possible underlying important factors.

The findings, while not new, reinforce the importance of the timely assessment of skin integrity and identification of any pressure damage so that appropriate pressure-relieving intervention can be initiated, especially within the frail older person group [17]. The findings suggest that there are several factors that when viewed in isolation may not provide a comprehensive account of the situation. However, when these factors are considered collectively, they offer some hypotheses for further analyses that might account for the increasing incidence of category 2 and 3 pressure ulcers. 

The evaluation suggests that those who developed a pressure ulcer during this specific period were primarily ‘frail’ older people aged between 80–90 years, many of whom experienced extended waits within A&E and between three and four moves before the development of the pressure ulcer. Brown-O’Hara (2014, p. 8) [18] highlights that this particular group are most at risk when she states that “the five conditions most commonly considered geriatric syndromes are: pressure ulcers, incontinence, falls, functional decline and delirium. Malnutrition, eating and feeding problems, sleeping problems, dizziness and syncope and self-neglect have also been classified as geriatric syndromes.” The fact that this group developed hospital-acquired avoidable pressure ulcers stresses the importance for extra vigilance and interventions to prevent pressure damage. Bianchi and Cameron, (2008) [19] stress the importance of a systematic approach to skin assessment. This should be timely, meaning the reporting of pressure ulcers becomes key considering revised requirements for pressure ulcer reporting (NHSI, 2018) [7].

Whilst these findings cannot be used to predict ‘risk’ at this point, they do provide some valuable insights and possible reasons for the increase in prevalence of avoidable pressure ulcers during this time span that can be used to inform a more formal piece of research, which would facilitate the development of a predictive model to isolate those factors which were most significantly associated with the development of pressure ulcers. Other important factors observed included the length of time individuals spend in A&E, with many experiencing 15–20 h waits. The situation is further compounded when one considers the type of surface individuals are nursed on—primarily an A&E trolley. While one of these factors may have a detrimental impact on skin integrity, collectively, they exacerbate the situation, creating a critical combination of factors resulting in pressure damage.

Amongst this cohort, the most common site for the development of a pressure ulcer was the heels and buttocks and the size of the pressure ulcer varied greatly with many of these being small. It is hypothesised that this combined with the number of ward transfers may result in a delay in the detection and recording of the pressure damage. While this is speculative, it does offer a plausible explanation for the increase in hospital-acquired avoidable pressure ulcers. If a thorough assessment of pressure areas is not undertaken within the first admission portal, then this may set in action a chain or sequence of events that place the individual at greater risk. 

The delayed reporting of pressures in A&E and in other clinical areas throughout the admission process may be associated with the failure to achieve a skin inspection and implement timely pressure area intervention. This cycle or chain of events must be broken with the timely implementation of pressure-relieving interventions. This may account for the delayed manifestation of the pressure ulcer in terms of the number of days before its appearance. 

From this work, we can conclude that a number of factors appeared common amongst those who developed a pressure ulcer and should be considered and mitigated when caring for older people at all times but especially during periods of escalation such as those experienced during ‘winter pressures’. These factors include age, with those particularly at risk aged 80+ years. In addition, females were observed to be have more pressure ulcer damage. However, this might just reflect the fact that more women are likely to fall into this age category than men. This may reflect current trends in life expectancy, with women living longer than men. The difficulty is that only those who developed a pressure ulcer were included and so conclusions about causal relationships cannot be made at this stage. The investigation has, however, given valuable information for further work.

## 5. Conclusions/Recommendations

### 5.1. Improvements to Date

- Redesign of A&E documentation to focus on preventative measures where operational pressures delay opportunity to perform a full risk assessment/body map, etc. The admission documentation now has an ‘Immediate Interventions’ section, guiding staff to work on the assumption of risk for all patients nursed on trolleys.
- Review of emergency portal access to heel offloading devices, beds and dynamic mattresses to ensure that they have adequate supply to meet increases in demand.
- AMU are currently undertaking a trial involving Acute Medical Rapid Assessment (AMRA) involving staff from AMU working with colleagues in A&E with the view to speed up the assessment process and reduce the number of hours in A&E, moves and admissions.
- Tissue Viability training and support provided on key themes and as part of the yearly statutory and mandatory training within the emergency portals.
- Dissemination of the key themes emerging from the RCA’s of avoidable pressure ulcers during this period—Trust-wide via the ‘Wound Wednesdays’ and TV ‘Tips on Tuesday’ Staff education sessions.
- Established a Pressure Ulcer Strategy Group that is multidisciplinary comprising representation from ambulance service, portering, nursing assistants, tissues viability, quality, medicine, AMU, allied health professionals, all in-reach services, Commissioners, NHS England and R&D. The purpose of the group will be to review and develop a targeted pressure ulcer prevention strategy for our admission portals.

### 5.2. Short Term

- Review A&E nursing assessment for the risk of pressure ulcer development and implementation of a relevant and targeted action plan (skin bundle/comfort rounds)/access to heel offloading devices/access to beds and dynamic mattresses in the department (review current SOP).
- Reinforce the importance of early intervention and the utilization of all pressure-relieving equipment available, especially with A&E and other emergency portals so that the chain of event identified in this evaluation can be broken.
- Raise awareness of the specific factors that appear to predispose particular groups of individuals, particularly those who are frail, over the age of 80–90 years, who spend an extended length of time in A&E and experience multiple moves.
- Review the process in A&E for supporting older people, especially those diagnosed with ‘frailty’, ensuring preventative action taken, shortest possible stay and quick transfer from A&E with minimal moves (maximum 1) to their final destination of care.

### 5.3. Long Term

- Conduct a more formal piece of research, using a larger representative dataset, incorporating a wider number of variables such as staffing, so that comparisons between those who developed a pressure sore during this period and those who did not can be undertaken to inform a predictive model.
- Develop a research protocol and seek the necessary Health Research Authority approval to access the relevant data sets within UHNM and the necessary authorization and permissions to conduct the investigation within the organization.
- Utilize more advanced parametric statistical tests (such as regression analysis) to develop a predictive model of those factors that may contribute to the development of a pressure ulcer during times of escalation and reduced capacity within the organization.

## Figures and Tables

**Table 1:** Key definitions.

Definition of a Pressure Ulcer NHS Improvement (2018) [7]	“A pressure ulcer is localised damage to the skin and/or underlying tissue, usually over a boney prominence (or related to a medical or other device), resulting from sustained pressure (including pressure associated with shear). The damage can be present as intact skin or as an open ulcer and may be painful”.
Avoidable Pressure Ulcer (Wound, Ostomy and Continence Nurses Society, 2009) * [8]	“Avoidable” means that the person receiving care developed a pressure ulcer and the provider of care did not do one of the following: evaluate the person’s clinical condition and pressure ulcer risk factors; plan and implement interventions that are consistent with the persons needs and goals, and recognised standards of practice; monitor and evaluate the impact of the interventions; or revise the interventions as appropriate.” UHNM policy is that any category 3 or 4 pressure ulcer that developed within 72 h of admission would be considered a deep tissue injury present on admission.

* It should be noted that under the revised guidance from NHSI (National Health Service Improvement) from 1st April 2019, the terms avoidable/unavoidable would no longer be used. However, learning for improvement at UHNM will continue with reference to evidence of effective care.

**Table 2:** Overview of the demographics and admission variables included in the evaluation.

Demographics/Variables	Male		Female		Total	
Count	18		19		37	
A&E attendance	15		17		32	
Admission body map completed	10		12		22	
A&E trolley	11		15		26	
-	**Mean**	**Std Dev**	**Mean**	**Std Dev**	**Mean**	**Std Dev**
Age (in years)	83.5	5.38	82.36	9.52	82.95	7.703
Number of moves	2.12	0.93	2.69	1	2.4	1
Hours in A&E	9.05	7.83	11.83	7.46	10.4	7.67
Total hours in emergency portal	32.3	63.9	43.2	56.6	37.9	59.7
Timespan to Pressure Ulcer development from admission (days)	17.1	21.24	19.68	18.68	18.43	19.63

**Table 3:** Time between A&E admission and body map/skin bundle. (35 patients * 2 patients had no A&E admission).

Time Between A&E Admission and Body Map/Skin Bundle	Number of Patients
Within four hours	8
Patients with heels offloaded	1
More than four hours	9
No skin bundle completed	8
No scanned Emergency Department (ED)notes	9
TOTAL	35

**Table 4:** Frequency of pressure ulcers.

Gender	Category 2		Category 3		Total	
Male	9	(47.4%)	9	(50%)	18	(48.6%)
Female	10	(52.6)	9	(50%)	19	(51.3%)
Total	19	(100%)	18	(100%)	37	(100%)

A Chi-square test of independence was calculated comparing the frequency of pressure ulcer categories in males and females. No significant interaction was found (X^2^ (1) = 0.026, *p* = 0.873).
